# Individual Impact of Distinct Polysialic Acid Chain Lengths on the Cytotoxicity of Histone H1, H2A, H2B, H3 and H4

**DOI:** 10.3390/polym9120720

**Published:** 2017-12-16

**Authors:** Kristina Zlatina, Thomas Lütteke, Sebastian P. Galuska

**Affiliations:** 1 Institute of Reproductive Biology, Leibniz Institute for Farm Animal Biology (FBN), Wilhelm-Stahl-Allee 2, 18196 Dummerstorf, Germany; zlatina@fbn-dummerstorf.de; 2 Institute of Veterinary Physiology and Biochemistry, Justus-Liebig-University, Frankfurter Str. 100, 35392 Giessen, Germany; thomas.luetteke@vetmed.uni-giessen.de

**Keywords:** polysialic acid, histones, neutrophil extracellular traps

## Abstract

Neutrophils are able to neutralize pathogens by phagocytosis, by the release of antimicrobial components, as well as by the formation of neutrophil extracellular traps (NETs). The latter possibility is a DNA-meshwork mainly consisting of highly concentrated extracellular histones, which are not only toxic for pathogens, but also for endogenous cells triggering several diseases. To reduce the negative outcomes initiated by extracellular histones, different approaches like antibodies against histones, proteases, and the polysaccharide polysialic acid (polySia) were discussed. We examined whether each of the individual histones is a binding partner of polySia, and analyzed their respective cytotoxicity in the presence of this linear homopolymer. Interestingly, all of the histones (H1, H2A, H2B, H3, and H4) seem to interact with α2,8-linked sialic acids. However, we observed strong differences regarding the required chain length of polySia to bind histone H1, H2A, H2B, H3, and H4. Moreover, distinct degrees of polymerization were necessary to act as a cytoprotective agent in the presence of the individual histones. In sum, the outlined results described polySia-based strategies to bind and/or to reduce the cytotoxicity of individual histones using distinct polySia chain length settings.

## 1. Introduction

Besides other mechanisms, neutrophil extracellular traps (NETs) serve to combat pathogens, such as fungi, viruses, and bacteria. For this purpose, NETs are a meshwork of antimicrobial peptides (AMP), chromatin, and enzymes [1]. Histones that are located in the chromatin are also described to function as AMPs [2,3]. The linker histone H1, as well as the four core subunits H2A, H2B, H3, and H4 possess—like other AMPs—a positive charge and are able to bind and penetrate negatively charged cell membranes [4,5]. This ability is not only toxic for invaders, but also for host cells [6]. Interestingly, Urban and coworkers demonstrated that histones are the most abundant proteins in NETs and the concentration is significantly higher for H2A (26.29%) and H2B (23.95%), representing approximately 50% of the total protein fraction [7]. As already well known, histones play important roles beyond the nucleus [8]. Besides the direct cytotoxic characteristics of extracellular histones, an induction of the immune response by free extracellular histones was shown via Toll-like receptors (TLRs) TLR2 and TLR4 [9]. TLR9 instead is activated by extracellular DNA [10], and also triggers an immune response [11]. An insufficient degradation by DNases and subsequent removal of NETs through macrophages [12], or an excessive NET-formation, may lead to production of antibodies against histones and DNA [13], as well as to an increased activity of these pattern recognition receptors [14]. This may lead to autoimmune diseases, like systemic lupus erythematosus (SLE) [15]. DNA and histones that originated from an excessive NET formation are further associated with the development of inflammatory disorders such as thrombosis, cystic fibrosis and sepsis [16,17,18,19]. The listed examples indicate that histones are not just important for the packaging of DNA, but also belong to the innate immune system. Thus, it is of interest to identify molecules that bind histones and affect their extracellular activities.

Previous studies already showed cytoprotective effects of the polysaccharide polysialic acid (polySia) against NETs and the histones that are contained in them [20,21]. These polymers consist of α2,8-linked *N*-acteylneuraminic acid residues, and are especially involved during the development of the brain and several other organs [22,23,24]. However, polySia is also present during processes of the immune system modulating, for instance, migration processes [25,26,27,28,29,30], and are often in direct neighborhood with NETs [21,31]. Although it is already known that polySia can inactivate the cytotoxic characteristics of histone-complexes in a chain length dependent manner [32], and can be used as a molecular anchor to accumulate nanoparticles on NET fibers [33], it is unknown, which one of the five histones is targeted by polySia. Until now, only undefined histone-mixtures originated from in vitro generated NETs or thymus were used [20,21,32,33] including e.g., histone monomers up to octamers—as well as numerous different epigenetic modifications [34]. 

In the present study, we examined the cytotoxicity of each individual histone in connection with a polySia application. The histones that are used in the present study were recombinantly expressed in bacteria and lack any eukaryotic specific posttranslational modification of histones. They were applied as model-proteins to get information about the interaction between polySia and the “naked” protein backbones of the individual histones. To this end, we studied the ability of polySia with different degrees of polymerization (DP) to bind these distinct histones. The observed results demonstrated several differences in the binding capacity of polySia to the individual histones, as well as in the cytoprotective capability of polySia against the histone-mediated cytotoxicity.

## 2. Materials and Methods

### 2.1. Materials 

For cell culture experiments, LPS was removed from colominic acid (Gerbu, Heidelberg, Germany) using C18 cartridges (Thermo Fisher Scientific, Dreieich, Germany), according to manufacturer specifications, as already mentioned in [33]. Used histones were: histone from calf thymus (Sigma-Aldrich, Steinheim, Germany) and recombinant human H1, H2A, H2B, H3.1, and H4 (New England Biolabs, Frankfurt am Main, Germany). All of the reagents used were of analytical grade.

### 2.2. Binding Model

The model was generated as previously described in detail [33]. The histone complex of Protein Data Bank (PDB) entry 3wa9 (PMID: 24311584) was used. The interaction of the histone complex with four polySia chains was modeled by YASARA. Different colors were attributed to the individual histones: H2A—green, H2B—cyan, H3—orange, H4—yellow. All four polySia chains are colored purple, numbered, and indicated by arrows. The location of DNA in the histone-DNA complex is shown as cords.

### 2.3. Fractionation and Determination of Sialic Acid Polymers by HPLC 

For cell culture and native agarose gel experiments, 10 mg colominic acid (Gerbu) were separated and collected according to the degree of polymerization (DP 3–15; 15–23; 23–34; 34–46; >46), as described in detail in [32,33]. The eluents were milliQ water (E1) and 4 M ammonium acetate buffer (E2) at a flow rate of 2.5 mL/min with following gradient using a DNAPac PA-100 (22 × 250 mm) (Thermo Fisher Scientific): t0 min = 0% (*v*/*v*) E2, t20 min = 13% (*v*/*v*) E2, t30 min = 17% (*v*/*v*) E2, t45 min = 19% (*v*/*v*) E2, t85 min = 21% (*v*/*v*) E2, t110 min = 100% (*v*/*v*) E2. The DP of fractionized polySia was controlled, as described previously [32]. The same eluents were also used for the analytical DNAPac PA-100 column (22 × 250 mm) (Thermo Fisher Scientific). Following gradient with a flow rate of 1 mL/min was used: t0 min = 0% E2; t5 min = 0% E2; t15 min = 8% E2; t20 min = 11% E2; t35 min = 14% E2; t55 min = 16% E2; t100 min = 20% E2; t130 min = 23% E2, and t131 min = 100% E2.

### 2.4. Quantification of Sialic Acid Polymers by HPLC 

To determine the concentrations of fractionated sialic acids, the samples were hydrolyzed and labeled with 1,2-diamino-4,5-methylenedioxybenzene (DMB), as already described [35,36,37]. For hydrolysis, samples were dissolved in 0.2 M trifluoroacetic acid (TFA) for 4 h at 80 °C and dried. In the next step the samples were dissolved in 80 μL DMB-reaction buffer (9 mM sodium hydrosulfite, 0.5 M β-mercaptoethanol, 20 mM TFA, 1.35 M DMB) and were incubated at 55 °C for 2 h to allow for the DMB-labeling. The addition of 20 µL 0.2 N NaOH stopped the reaction. The final DMB-labeled fractions were analyzed via Superspher 100 C-18 column (250 mm × 40 mm, Merck-Hitachi, Darmstadt, Germany) at 40 °C by high performance liquid chromatography (HPLC) (Smartline System, Knauer, Berlin, Germany), as described in detail in [38].

### 2.5. Cell Culture Experiments

5B8 cells were cultured in Roswell Park Memorial Institute (RPMI) medium (Gibco, Darmstadt, Germany), including 10% (*v*/*v*) fetal calf serum (FCS; Thermo Fisher Scientific, Dreieich, Germany) and 1% Streptavidin at 37 °C and 5% CO_2_. 30,000 cells were seeded per well on 96-well plate in 100 µL of RPMI medium, including histones or polySia, and were incubated for 1 h 40 min at 37 °C and 5% CO_2_ [32,33]. Here, the concentration of histones was 60 µg/mL, and the concentration of Neu5Ac or fractionated sialic acid polymers was 40 µg/mL. To determine the cytotoxicity a lactate dehydrogenase (LDH) cytotoxicity assay (BioVision, Milpitas, CA, USA) was applied.

### 2.6. Agarose Gel-Electrophoresis 

Histones, polySia, and mixtures of histones with Neu5Ac or fractionated sialic acid polymers were incubated in 50 mM Tris for 1 h at 30 °C. 1 µL glycin was added and samples were loaded on a 2% agarose gel (peqLab, Erlangen, Germany) in 500 mM Tris/HCl, 160 mM boric acid, 1 M urea, pH 8.5. The electrophoresis was performed with a running buffer (90 mM Tris/HCl, 90 mM boric acid, pH 8.5) at 80 V for 5–6 h (modified according to [39,40]). The proteins were stained with roti-blue (Roth, Karlsruhe, Germany), according to manufacturer specifications.

### 2.7. Statistical Analysis 

Data sets were statistically evaluated by Student’s *t* test (unequal variances, two-tailed) by using Microsoft Excel. Significant differences are given: n.s. (not significant), *p* > 0.05; * *p* < 0.05; ** *p* < 0.01; *** *p* < 0.001; **** *p* < 0.0001.

## 3. Results & Discussion

### 3.1. Binding Model between PolySia and Histones 

In a previous study [32], we already used a model of a histone-DNA-complex (Protein Data Bank entry 3wa9) and four polySia chains with a chain length of 20 sialic acid units to get a hint for possible binding areas between polySia and histone octamers. However, it was not possible to distinguish between the different histones of this octamer using the old model. Thus, the individual interaction areas of H2A, H2B, H3, and H4 could not be located until now.

**Figure 1:**
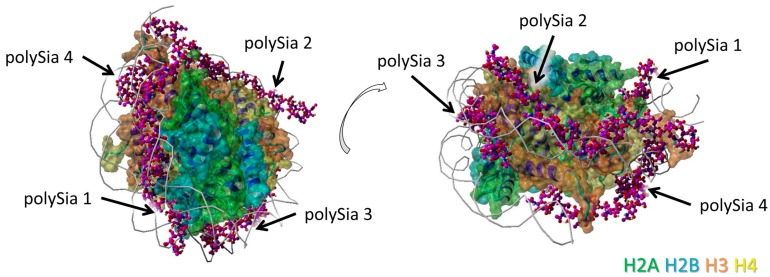
Model of interaction between polySia and histones. This model includes the histone-octamer of the Histone–DNA complex (Protein Data Bank entry 3wa9) as receptor and four polySia chains of 20 sialic acid units as ligands. The polySia chains are numbered and indicated by arrows. The two parts show the model from two different perspectives (rotated as shown by the open arrow). Color-code: H2A—green, H2B—cyan, H3—orange, H4—yellow, polySia—purple, DNA—gray (as cords).

For the current study, this model was re-evaluated to examine the role of individual histones in binding of polySia. For this purpose, the individual histones are colored differently (Figure 1). The model is shown from different perspectives for a better visualization of all the binding sites between polySia chains and the histones.

**Figure 2:**
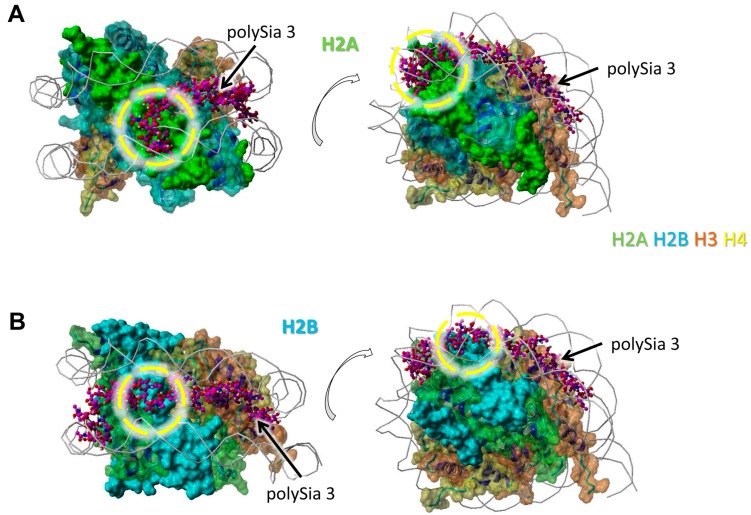
Model of interaction between polySia and H2A as well as H2B. The model was generated as described in Figure 1. Color-code: H2A—green, H2B—cyan, H3—orange, H4—yellow, polySia—purple, DNA—gray (as cords). Potential binding areas are highlighted by dashed circles for the histones; (**A**) Histone H2A, (**B**) Histone H2B. The numbered polySia chains are indicated by arrows. Both models are shown from two different perspectives (rotated, as shown by the open arrows).

We observed a particularly close neighborhood of eight successively linked sialic acid residues with H2A (Figure 2A). Here, polySia is partially colocalized with the winding of the DNA. The same polySia chain binds to the directly neighbored H2B with five successive sialic acid units. The binding area of these residues is located between those of two DNA helices (Figure 2B). 

**Figure 3:**
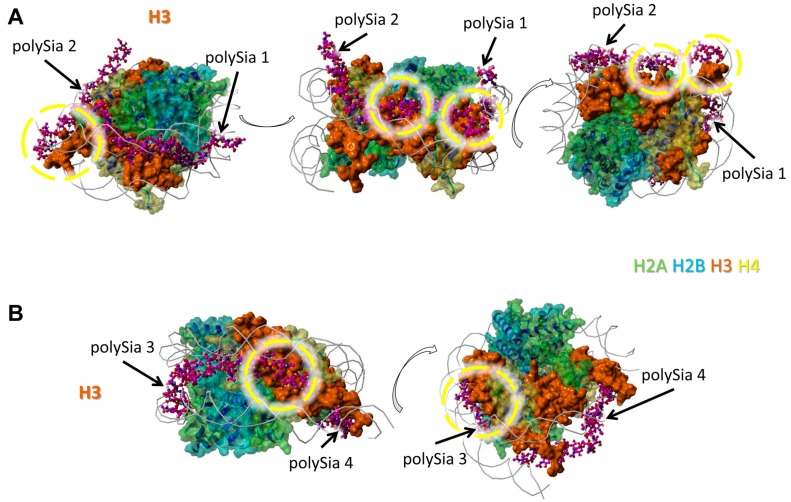
Model of interaction between polySia and H3. The model was generated as described in Figure 1. Color-code: H2A—green, H2B—cyan, H3—orange, H4—yellow, polySia—purple, DNA—gray (as cords). Potential binding areas are highlighted by dashed circles for the histones. The numbered polySia chains are indicated by arrows; (**A**) polySia chain 1 and 2, (**B**) polySia chain 3 and 4. The model is shown from five different perspectives (rotated as shown by the open arrows).

All four modeled polySia chains seem to bind histone H3. However, just one or two continually linked sialic acid units of these polySia chains are in close neighborhood to H3. In Figure 3A we show two of four polySia chains and in Figure 3B the remaining two. The binding areas are partially colocalized with those of DNA. 

**Figure 4:**
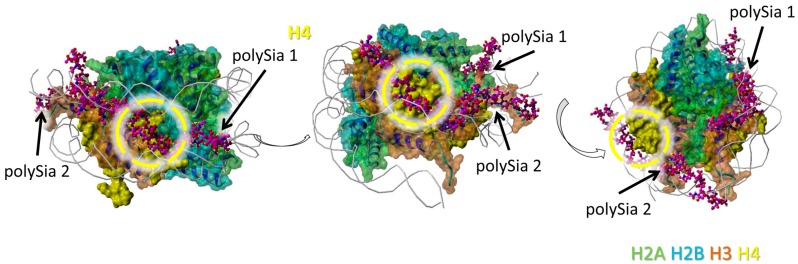
Model of interaction between polySia and H4. The model was generated as described in Figure 1. Color-code: H2A—green, H2B—cyan, H3—orange, H4—yellow, polySia—purple, DNA—gray (as cords). Potential binding areas are highlighted by dashed circles for the histones. The numbered polySia chains are indicated by arrows. The model is shown from three different perspectives (rotated as shown by the open arrows).

H4 seems to interact with two polySia chains in total. The model shows only short distances for possible interactions between scattered sialic acid residues of the polySia chains (and not a cluster of directly linked sialic acid residues) and the protein backbone (Figure 4).

Nevertheless, regions of all histones are covered by polySia, when H2A, H2B, H3, and H4 are present in a histone octamer. However, it has to be kept in mind that apart from these, there might be further possible interaction areas for polySia with the individual histones that are hidden by other histones as long as they build complexes. In addition, based on the variable length and flexibility of polySia there might be more potential binding motives for polySia than shown in Figure 1, Figure 2, Figure 3 and Figure 4.

### 3.2. PolySia Interacts with Individual Histones

In our previous study [21,33], we demonstrated the influence of different concentrations and DPs of polySia on its binding capacity to undefined complex mixtures of different histones that originated from in vitro generated NETs and/or thymus samples. In contrast to this, in the present study, we aimed to figure out how polySia influences the characteristics of the individual histones using recombinantly expressed H1, H2A, H2B, H3 and H4. For this purpose, we investigated the binding capacity of polySia to histones by native agarose gel electrophoresis in a first set of experiments. The positively charged histones migrate to the negative pole under these conditions, based on their basic character (Table 1).

**Figure 5:**
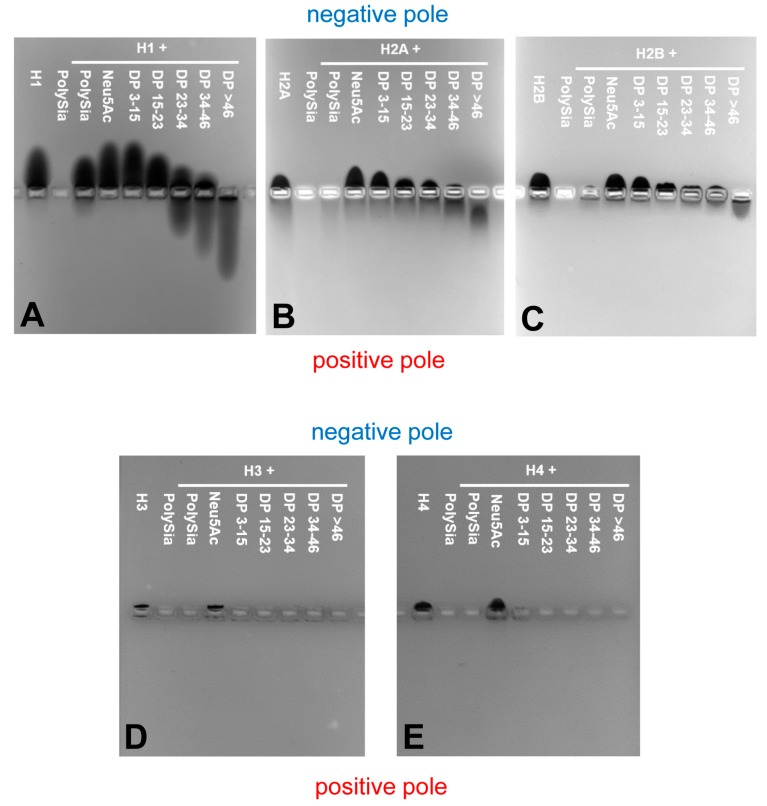
Non-covalent binding assays. PolySia interacts with individual histones in a degree of polymerization (DP)-dependent manner and causes a migration-shift in native gel electrophoresis. Proteins (5 µg) and a mixture or defined DP of polySia (2.5 µg) were incubated, electrophoretically separated and stained with Coomasie Blue. (**A**) Histone H1; (**B**) Histone H2A; (**C**) Histone H2B; (**D**) Histone H3; (**E**) Histone H4. Representative gels of three independent experiments.

Successively, we incubated the individual histones with different DP of polySia, loaded them on native gels, and stained the proteins with Coomassie Blue. If the negatively charged polySia and positively charged histones interact with each other, then this interaction should result in a migration shift of histones to the positive pole. Interestingly, all of the histones showed individual migration characteristics (Figure 5).

The “linker” histone H1 (Figure 5A) migrates, as expected, to the negative pole. H1 seems to be a binding partner of polySia, as also previously described by Mishra et al. [41], and DPs higher than 23 led to a significant migration shift of H1.

Histone H2A and H2B show almost equal migration patterns (Figure 5B,C). Their migration is already influenced by polySia chains consisting of 15–23 sialic acid residues.

Histone H3 and H4 (Figure 5D,E) are showing the shortest migration distance. The missing staining of Coomassie Blue in case of H3 and H4 with DP > 1 let us assume that the proteins build directly complexes with short chains, e.g., dimers, as well as oligo- and polymers, which cannot be resolved by native gel electrophoreses.

Thus, the migration capacity of all the individual histones can be influenced by distinct chain lengths of sialic acid polymers.

### 3.3. Cytotoxicity Effects Are Histone-Dependent

**Figure 6:**
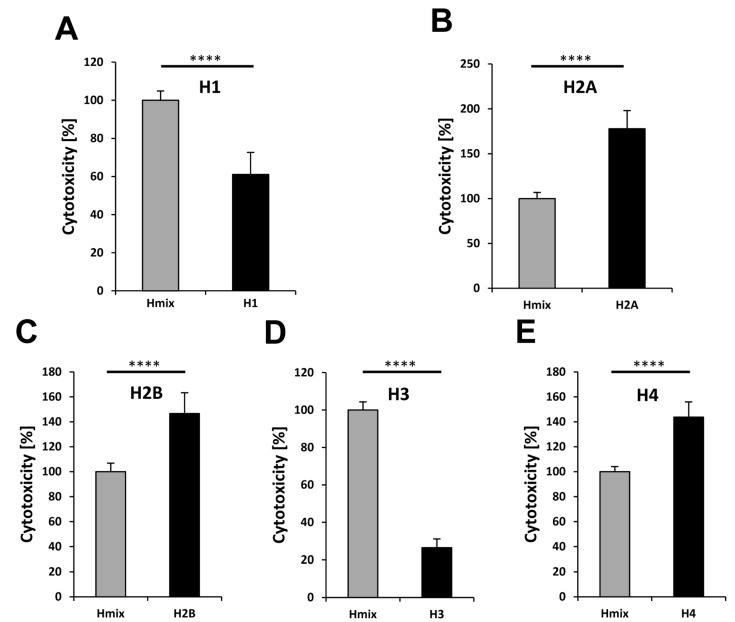
Cytotoxicity of individual histones compared to a mixture of histones. The cytotoxic effects of the histone mix (60 µg/mL) and individual histones (60 µg/mL) on 5B8 cells are shown. The histone-mix (gray bar) was defined as 100%. (**A**) Histone H1; (**B**) Histone H2A; (**C**) Histone H2B; (**D**) Histone H3; and, (**E**) Histone H4. All of the experiments were performed three times independently. The statistical analysis was performed by *t*-test using Microsoft Excel: n.s., not significant; * *p* < 0.05; ** *p* < 0.01; *** *p* < 0.001; **** *p* < 0.0001.

To examine the cytotoxicity of the individual histones, we performed cell-culture experiments. To this end, cells were treated with the individual histones (Figure 6). In parallel, we incubated cells with a mixture of histones defining this examined cytotoxicity level as 100%. The results show that H1 and H3 are significantly less cytotoxic than the histone mix (Figure 6A,D). The observation of H1’s low cytotoxicity corresponds with previous studies [7,42]. Interestingly, H1 could not be detected in NETs until now [7,42]. Probably, H1 is the first released and/or degraded histone of all, enabling the decondensation of the nucleosomes.

In contrast to H1 and H3, histone H2A, H2B, and H4 showed a much higher cytotoxicity when compared to the histone mix (Figure 6B,C,E).

### 3.4. The Cytotoxicity of Histone H1, H2A, H2B, H3, and H4 Is Individually Influenced by PolySia

Table 1 lists the relative amounts of individual histones in NETs, where H2A and H2B representing with up to 72% the main histone fraction (percentages were calculated using values from [7]). Their high presence and cytotoxicity make H2A and H2B to important targets in fighting the negative outcomes of NETs. We examined to which extent polySia influences the cytotoxic properties of individual histones, and determined the required chain length of polySia. For this purpose, we incubated cells with the individual histones, as well as polySia with or without defined chain lengths.

**Figure 7:**
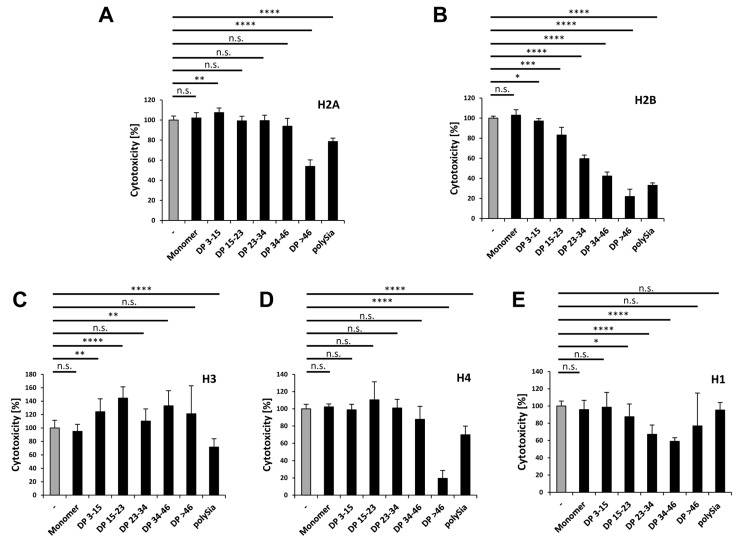
PolySia reduces the cytotoxicity of all histones. The cytotoxicity was determined for cells treated with each individual histone (60 µg/mL) alone or together with different DPs of polySia (40 µg/mL). The histone mediated cytotoxicity (gray bar) was set to 100%. All effects in presence of polySia are shown in black bars. (**A**) Histone H2A; (**B**) Histone H2B; (**C**) Histone H3; (**D**) Histone H4; and, (**E**) Histone H1. All of the experiments were performed three times independently. The statistical analysis was performed by *t*-test using Microsoft Excel: n.s., not significant; * *p* < 0.05; ** *p* < 0.01; *** *p* < 0.001; **** *p* < 0.0001.

In the case of H2A, only polySia chains consisting of more than 46 sialic acid residues reduce the histone-mediated cytotoxicity by up to 50% (Figure 7A). Based on the results of our migration studies using native gels, demonstrating that a DP of more than 15 units lead to migration shifts, we speculate that polySia mainly binds to those domains of H2A, which do not directly influence its cytotoxicity. However, the flexible free unbound part of long polySia chains might influence its integration into the biological membrane in a negative way.

H2B shows a pattern in chain length-dependent reduction of cytotoxicity, as mediated by polySia (Figure 7B) that was comparable to that of the histone mixtures published in an earlier study [32]. The cytoprotective effects of polySia on H2B reflect the results of our migration studies. Both of the experiments show the migration shift and the reduction of cytotoxicity by using increased DPs of polySia. This leads to the conclusion that polySia binds prevalently domains of H2B, which are responsible for the cytotoxic effect of this histone.

Besides H2A (38.02%) and H2B (35.63%), H3 represents the third biggest group (20.97%) of the histone fraction in NETs. Surprisingly, the addition of different polymer chain lengths of polySia to H3 resulted partially in increased cytotoxic values or had no effect (Figure 7C). However, unfractionated polySia decreased the cytotoxicity by up to 30% (Figure 7C).

Like H2A and H2B, histone H4 has a higher cytotoxicity than the histone mix (Figure 6E), but is almost absent (4.4% of all histones) in NETs (Table 1). Its cytotoxicity was only reduced by polySia with a DP >46 by up to 80% (Figure 7D). Surprisingly, this effect was reduced when unfractionated polySia was applied. Thus, shorter chains may prevent the binding of the more effective polySia chains consisting of more than 46 sialic acid residues.

Although histone H1 is absent in NET-fibers, the outlined experiments were also performed with H1. Here polySia chains with a DP higher than 14 were able to significantly reduce these negative effects (Figure 7E).

**Figure 8:**
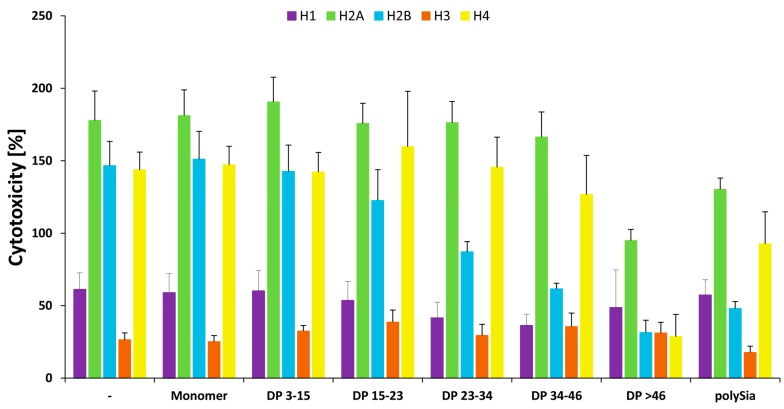
Summary of polySia’s influence on all histones in comparison to a mixture of all histones. In contrast to Figure 7 histone-mix mediated cytotoxicity was set to 100% and compared with all individual histones. All experiments were performed three times independently.

As summarized in Figure 8, the cytotoxic character of all the histones can be generally influenced by polySia. However, several differences were observed in the required chain length patterns. Thus, distinct chain lengths or chain length compositions have to be used depending on the target-histone.

## 4. Conclusions

In the outlined study, we demonstrated that sialic acid polymers bind to all of the individual histones. However, its binding capacity for the individual histones depends strongly on the DP. Interestingly, polySia is also able to influence the cytotoxic outcomes of all histones by the use of a distinct DP and/or a mixture of different chain length. The results strongly suggest that polySia influences the histone-cytotoxicity not just on the basis of charge alone, since the defined chain length has to be reached to modulate the cytotoxicity and/or the binding of histones. Thus, for each histone, an individual set of polySia chain length can be used to address the binding and/or cytotoxic effects of distinct histones. 

For instance, it is conceivable that polySia can be used to accumulate histones on biomedical surfaces without an inactivation of its antimicrobial function. H2A is discussed to be used as a novel “antibiotic” [43,44]. Based on our finding it seems to be possible to accumulate H2A without influence to its toxic characteristics using polySia with a DP <46. Comparable strategies might also be possible with other histones, since all four core histones were already described as AMPs in pacific white shrimps [45] and oncorhyncin II, a proteolytic product of histone H1, was also suggested as an AMP in the skin of rainbow trout [46]. However, the antimicrobial and/or cytotoxic characteristics must be balanced against each other and probably defined polySia chain length settings might be helpful. Thus, the outlined results provide a further basis to develop polySia-histone based strategies for biomedical applications.

## Figures and Tables

**Table 1:** Overview of the individual histone properties and the respective percentage of all histones in NET.

	H1	H2A	H2B	H3	H4
Amino acids	231	154	149	161	122
pI	10.11	10.95	9.76	11.02	11.3
Mass [kDa]	20.73	13.99	13.79	15.27	11.24
Basic amino acids	66	30	31	33	27
Acidic amino acids	9	9	10	11	10
Relative amount of histone [%] ^1^	-	38.02	34.63	20.97	6.38

^1^ The relative amount of each individual histone is based on the values for the molar amounts of histones from [7].
